# Riboflavin lowers blood pressure in hypertensive people with the *MTHFR* 677TT genotype

**DOI:** 10.1186/2049-3258-72-S1-K2

**Published:** 2014-06-06

**Authors:** Helene McNulty, JJ Strain, Mary Ward

**Affiliations:** 1 Northern Ireland Centre for Food & Health, University of Ulster, Coleraine, BT52 1SA, UK

## 

Hypertension, defined as a systolic/diastolic blood pressure of 140/90 mmHg or greater, is estimated to carry a 3-fold increased risk of developing cardiovascular disease (CVD), while treating hypertension significantly reduces CVD events, and stroke in particular. Among the many risk factors involved, there is much recent interest in the role of genetic factors that might predispose to hypertension.

Evidence from genome-wide association studies has identified an association between blood pressure and the gene encoding the folate-metabolising enzyme, methylenetetrahydrofolate reductase (MTHFR), while recent meta-analyses of observational studies show an increased risk of hypertension in people homozygous for the 677C→T polymorphism in MTHFR. Riboflavin (vitamin B2) in the form of FAD acts as a cofactor for MTHFR and we have been studying its modulating role in relation to this polymorphism. The variant enzyme is known from molecular studies to become inactive as a result of having an increased propensity to dissociate from FAD, but our earlier work suggested that supplementation with low-dose riboflavin could stabilise MTHFR activity *in vivo* in homozygous individuals. In recent years we showed that CVD patients with the relevant *MTHFR* 677TT genotype (compared to CC or CT genotypes) had significantly higher blood pressure, and that blood pressure was highly responsive to riboflavin intervention, specifically in the TT genotype group [1]. Further investigations confirmed this gene-nutrient interaction in hypertensive patients (with and without overt CVD), and furthermore showed that the blood pressure lowering effect of riboflavin in the TT genotype group was independent of the number and type of antihypertensive drugs that they were taking [2].

Although the precise mechanism linking this polymorphism to hypertension remains to be established, it would appear that the biological perturbation that leads to higher blood pressure in individuals with the MTHFR 677TT genotype is modifiable by correcting the variant MTHFR enzyme through enhancing riboflavin status. Thus riboflavin, targeted specifically at this genetically at-risk group, may offer a personalized non-drug approach to managing hypertension.
